# Effect of Ethanol on Micro-Vessels Diameter and Prevention of Thrombosis

**DOI:** 10.29252/wjps.8.2.249

**Published:** 2019-05

**Authors:** Mojtaba Nasiri, Mohammad Hossein Kardar

**Affiliations:** 1 School of Medicine, Shahroud University of Medical Sciences, Shahroud, Iran;; 2 Department of Plastic Surgery, School of Medicine, Shahroud University of Medical Sciences, Shahroud, Iran

**Keywords:** Ethanol, Vessel, Diameter, Thrombosis, Rabbit

## Abstract

**BACKGROUND:**

Microvascular surgery is one of the most important parts of reconstructive surgery. In the present study, the effect of ethanol on microvascular diameter and prevention of thrombosis was evaluated.

**METHODS:**

Totally, 80 vessels including 40 arteries and 40 veins in right and left ears of 20 adult rabbits were enrolled. Seven days after injection of ethanol to rabbit ear vessel, vessel diameter and thrombosis rate post-iced saline challenge were documented and compared to normal saline injection in contralateral ear as a control group.

**RESULTS:**

Vessel diameter in both arteries and veins in ethanol group was significantly larger than normal saline control group, and patency rates due to preventive effect of ethanol were also significantly higher in the ethanol group after iced saline challenge.

**CONCLUSION:**

Pretreatment with ethanol can enlarge vessel diameter and play a preventive role on thrombosis after iced saline challenge.

## INTRODUCTION

Microvascular surgery is one of the most important parts of reconstructive surgery. After recent improvements in surgical knowledge and techniques, it became a reliable method for reconstruction of body defects, especially in head and neck and distal limb areas.^1^^-^^3^ The success rate for free tissue transfers by microsurgical methods has been reported to be more than 90%, even up to 99 percent success rates have been shown in replantations.^4^ Even the failure rate of free flaps is actually low, failure does occur and thrombosis is the most common cause.^5^ Thrombosis is a major complication could lead to complete loss of replanted free transferred tissue and even death of patient.^6^^,^^7^

There are numerous predisposing factors to thrombosis including microvascular spasm, inadequate vessel size for microanastomosis, severity and mechanism of injury, technical errors in surgery and comorbidities like infection and being smoker^8 ^intact vessels adequate flow, absence of vasospasm and surgical expertise that are all needed for a reliable replantation,^9^^,^^10^ preventive techniques and maneuvers for vasospasm and thrombosis including vasopressors, warming and antithrombotics and vasodilators.^11^^,^^12^

Ethanol has various effects on different body systems. Alteration in peripheral blood flow including increased blood flow to face, skin and extremities occurs as vasodilation in periphery and is common after ethanol use.^13^ In animal models, ethanol causes relaxation of smooth muscles in vessels wall by activation and induction of NO and prostaglandins.^14^ In several *in vivo* and *in vitro* studies, it was shown that ethanol lowered vascular tone and vessels spasm.^15^ Ethanol also prevents thrombosis by inhibition of platelet aggregation and increasing the levels of plasminogen activators.^16^ Ethanol also induces its vasodilation effect by increasing bioavailability of nitric oxide (NO) and decreasing endothelin-1 synthesis.^17^^,^^18^ The aim of this study was to evaluate the effect of ethanol on vessels diameter and prevention of thrombosis.

## MATERIALS AND METHODS

Twenty adult rabbits were involved in this double blind animal study. In the test group, 2 mL of 10% ethanol was injected subcutaneously near central posterior artery and marginal vein with a care not to directly damage the vessel. Two milliliter of normal saline similarly was injected as a control group in contralateral ear. Seven days after injection, the animals were anesthetized by 2% xylazine (0.15 mg/kg) intramuscularly, 2% acepromazine (0.4 mg/kg) and ketamine (2.5 mg/kg). Reinjection of anesthetic drugs was done after 40 minutes or as needed. After shaving and prepping the site, a careful transvers skin incision for vessel access was made by No. 15 blade. 

Meticulous dissection around central artery and marginal vein was done and a small fine razor below the vessel was used for diameter measurement. The extent of dissection in test and control groups was the same in all performed operations by a senior surgeon who was double blinded. Operations were randomized between right and left ears. After completion of one ear, the contralateral ear was undertaken immediately and a megapixel camera was used for documentation and measuring of vessel diameter. After measurement, both ear vessels were exposed to iced saline simultaneously for 1 minute. Ten minutes later, arteries and veins were opened and any bleeding or thrombosis was recorded. Complete hemostasis and skin closure were conducted and the rabbits were carefully monitored for recovery. Data analysis was performed by SPSS software (Version 18, Chicago, IL, USA) and mean, standard deviation and prevalence were determined for statistical description. The comparison between vessel diameter and thrombosis rate was performed by T-test. A *p* value <0.05 was statistically considered significant. 

## RESULTS

The mean arterial diameter was significantly (0.02) larger in ethanol group (1±0.39 mm), when compared to the control group (0.65±23 mm). The mean venous diameter was also significantly larger in ethanol group (1.2±0.3) in comparison to the control group (0.57±0.18) (*p*<0.001). The arterial thrombosis in both sides was visible in 3 rabbits (15%) (Table 1). In one rabbit’s ear (5%), arterial thrombosis after iced saline challenge was seen only in ethanol pretreated side and in 11 rabbits (55%) were noticed only in saline side. In 5 rabbits (25%), none of the arterial rabbit’s ears demonstrated thrombosis (Table 1). 

**Table 1:** Characteristics of the vessels in ethanol and normal saline pre-treated groups

**Variable**	**Ethanol (n=20)**	**Control (n=20)**	***p*** ** value**
Average arterial diameter (mm)	1±0.39	0.65±23	0.02
Average venous diameter (mm)	1.2±0.3	0.57±0.18	<0.001
Arterial thrombosis (n)	4	14	0.01
Venous thrombosis (n)	3	16	<0.001

The thrombosis rate was significantly (*p*=0.01) lower in alcohol ethanol group (20%) than in saline group (70%). Venous thrombosis was observed in three rabbits of both ethanol and saline sides (15%). In none of the rabbits, isolated venous thrombosis in ethanol side happened. In 13 rabbits (65%), thrombosis occurred only in saline control side, while the thrombosis rate after iced saline challenge was significantly (*p*<0.001) lower in ethanol group (15%) in comparison to the saline group (80%) (Table 1).

## DISCUSSION

Various etiologies could lead to vessel thrombosis and vasospasm is the major cause. Lowering the resistance against blood flow creates a hyperperfusion state for the free flap and prevents its rejection.^19^ In the present study, vessel diameter in both arteries and veins were larger in ethanol group when compared to the control group. Surprisingly, this effect and enlargement in vessel diameter was more significant in venous group than arterial group. Vasospasm is a well known phenomenon in arteries but like our previous study with botulinum toxin A,^1^ veins respond more to alcohol than arteries. 

In microvascular surgeries, vessel patency and diameter are clinical factors of operation.^20^ In our study, thrombosis rate in ethanol group was lower than control group and like the diameter of the vessels, the rate of venous thrombosis was greater than the arterial thrombosis. In the present study, alcohol was used and its chemical vasodilatory effect and by iced saline thermal challenge were evaluated and its effect on preventing thrombosis was determined. Janz *et al.* in an animal model examined the effect of botulinum toxin B on prevention of thrombosis induced by anastomosis following acute injuries.^21^

For induction of thrombosis, samples were subjected to vasospastic stress at 12, 24, 48, 72 and 120 hours after anastomosis. Vasospastic stress involves the iced saline challenge and systemic treatment with phenylephrine. After the vasospastic test, the wounds were opened and vascular diameter and thrombosis were examined. In this study like to ours, botulinum toxin B could prevent or reduce intravenous thrombosis at all times, except 120 hours after injection. This finding indicates to its acute influence on thrombosis after vascular anastomosis.

Also botulinum toxin Acan was shown to increase the vascular diameter and to decrease vascular thrombosis after anastomosis.^22^ The use of thrombolytics has shown similar results to our study, although the risk of bleeding following the use of these drugs increased, and did not affect the size of the vasculature.^23^ Unlike botulinum toxin and thrombolytics, alcohol was demonstrated not to have serious complications for the arteries and was considered safe. We used rabbit’s ears in our study. These vessels have several prominent advantages. First, they are superficial and have safe and reliable vascular access showing that the use of surface vessels is less confusing and increases the feasibility of studying.^22^

The second most important feature of the rabbit’s ears is the presence of cholinergic and adrenergic structures in the rabbit’s ear arteries, which makes it suitable for vasospastic models.^24^ The third is the presence of very rich collateral vessels in the rabbit’s ears that maintains the rabbit’s ears after vascular ligation. The advantages of our study were use of an appropriate sample size leading to more valuable statistical analysis and the use of ethanol without any lethal or dangerous effects on the vital organs and patient’s life. 

Our study had some limitations, despite all of the anastomoses performed by a microvascular surgeon who was blinded to the study and some of the thrombosis that might have been followed by technical errors during surgery. Our results on rabbit animal model are not necessarily similar to the findings of studies in human models. However, we believe that the use of ethanol in human models have important implications for the prevention of thrombosis and requires further trials. We demonstrated that use of ethanol resulted in an increase in arterial and venous diameters, which further leads to a reliable and sufficient perfusion flow in the tissue and transplanted flaps. Alcohol was shown to reduce vasospasm and the rate of thrombosis after transplantation of microvascular free flaps. As a result, alcohol could increase the survival rate of the transferred flap and reduce the rejection of the transplant.

## CONFLICT OF INTEREST

The authors declare no conflict of interest.
